# From Problem Taxa to Problem Solver: A New Miocene Family, Tranatocetidae, Brings Perspective on Baleen Whale Evolution

**DOI:** 10.1371/journal.pone.0135500

**Published:** 2015-09-02

**Authors:** Pavel Gol’din, Mette Elstrup Steeman

**Affiliations:** Department of Natural History and Palaeontology, The Museum of Southern Jutland, Lergravsvej 2, 6510, Gram, Denmark; New York Institute of Technology College of Osteopathic Medicine, UNITED STATES

## Abstract

Miocene baleen whales were highly diverse and included tens of genera. However, their taxonomy and phylogeny, as well as relationships with living whales, are still a subject of controversy. Here, *“Mesocetus” argillarius*, a poorly known specimen from Denmark, is redescribed with a focus on the cranial anatomy. It was found to represent not only a new genus, *Tranatocetus* gen. nov., but also a new family; Tranatocetidae. The whales of this family have the rostral bones either overriding or dividing the frontals; the rostral bones are contacting the parietals and nasals dividing the maxillae on the vertex; the occipital shield is dorsoventrally bent. The tympanic bulla is particularly characteristic of this family featuring a short, narrow anterior portion with a rounded or squared anterior end and a wider and higher posterior portion that is swollen in the posteroventral area. A phylogenetic analysis including 51 taxa supports a monophyletic group comprising most Neogene and modern whales, with Tranatocetidae being possibly closer related to Balaenopteridae (rorquals) than to Cetotheriidae. Tranatocetidae exhibit a charahteristic bulla shape. In fact, all Neogene and modern mysticete families examined have a unique shape of the tympanic bulla that is diagnostic at family-level. Inclusion of problematic taxa like *Tranatocetus argillarius* in phylogenies brings new understanding of the distribution and diagnostic value of character traits. This underlines the need for re-examination of earlier described specimens in the light of the wealth of new information published in later years.

## Introduction

The earliest toothless baleen whales originated approximately 30 million years ago [1]. Only 15 species exists today, but in the Middle and Late Miocene (16–5 million years ago, mya) the group was highly diverse comprising dozens of genera [2]. Recently, a renewed interest in the evolution of baleen whales has led to a wealth of new information with more than 40 species described in the last 15 years [3]. However, the taxonomy and phylogeny of Miocene baleen whales is still a subject of controversy. The phylogenies published within the last decade either strongly disagreed in the position of several groups or showed poor resolution within lineages [4–11]. Different researchers have suggested the extinct family of Cetotheriidae to be closely related to either the living gray whale *Eschrichtius robustus* or the pygmy right whale *Caperea marginata* [5, 8]. Controversies may be due to lack of data and taxa included in the analysis, so adding basal taxa may improve resolution and clarify relationships. The phylogenetic affinities of *“Mesocetus” argillarius* from the Late Miocene of Denmark [12] were discussed by Steeman [5] who found it to be a sister taxon to Cetotheriidae. The suite of characters displayed by *“Mesocetus” argillarius* may therefore play a key role in elucidating the origin of the modern baleen whales.

Here, *“Mesocetus” argillarius* is redescribed with focus on previously unpublished cranial anatomy. A novel idea of the evolution of Neogene and living baleen whales is presented based on a phylogeny including a combination of newly described species and species described during the 19^th^ and early 20^th^ centuries without the abundance of comparative material available today.

## Material and Methods

### Ethics statement

This study concerned only non-living, previously preserved museum specimens; therefore no permission from an Institutional Animal Care and Use Committee was needed; no permits were required for the described study, which complied with all relevant regulations.

**Fig 1:**
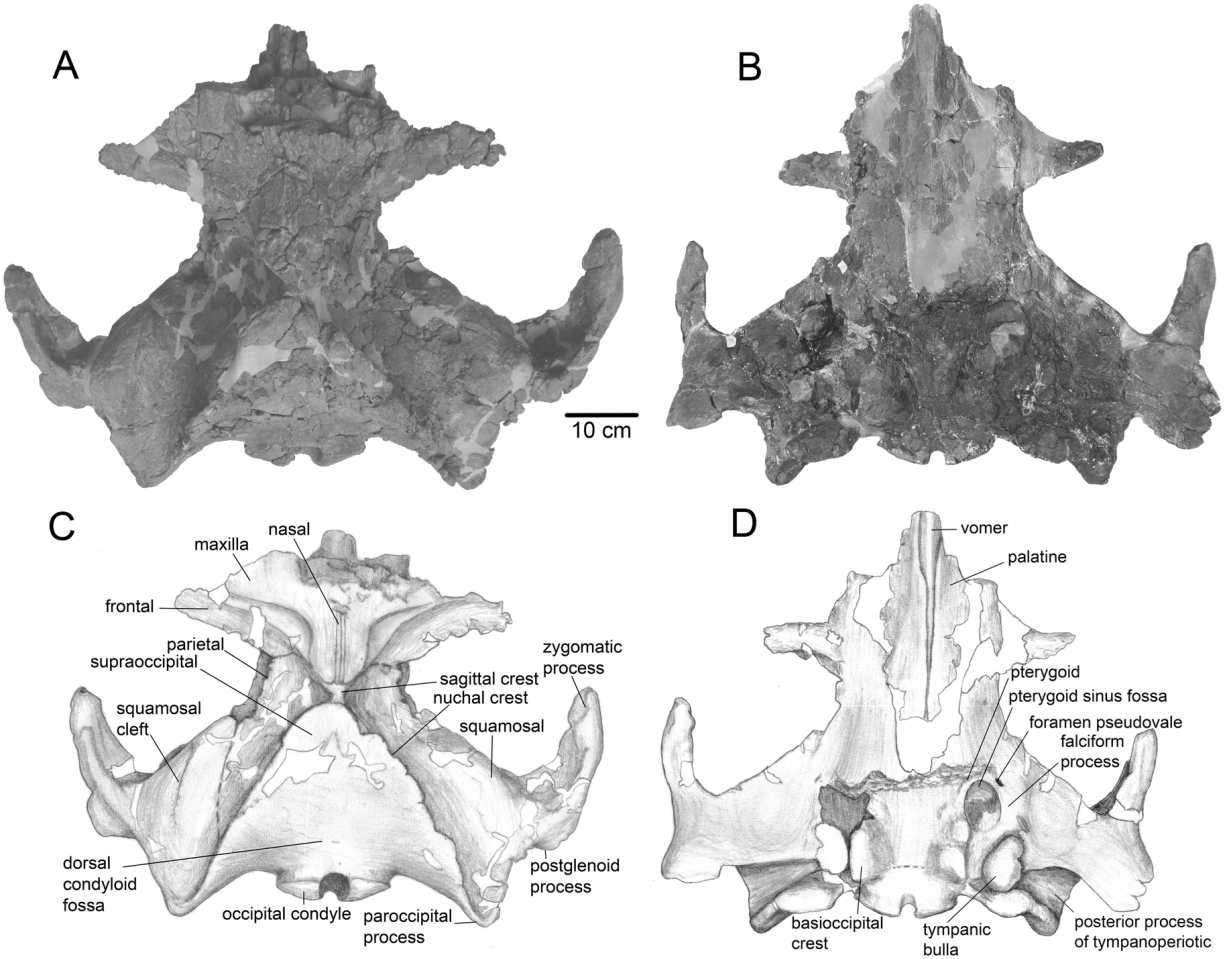
The skull of *Tranatocetus argillarius*, MGUH VP 2319. A, B, dorsal views; C, D, ventral views.

**Fig 2:**
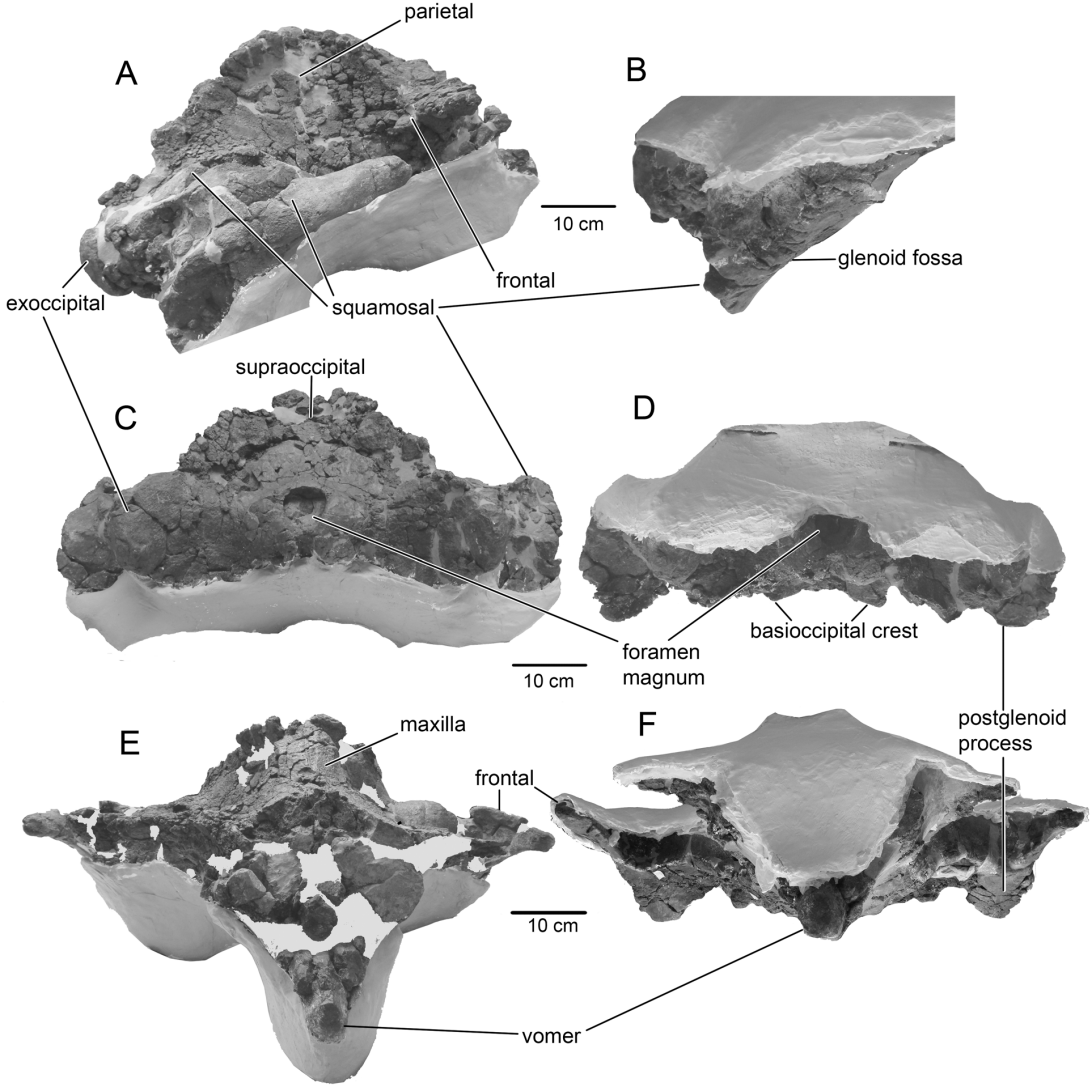
The skull of *Tranatocetus argillarius*, MGUH VP 2319. A, B, lateral view; C, D, anterior view; E, F, posterior view.

**Fig 3:**
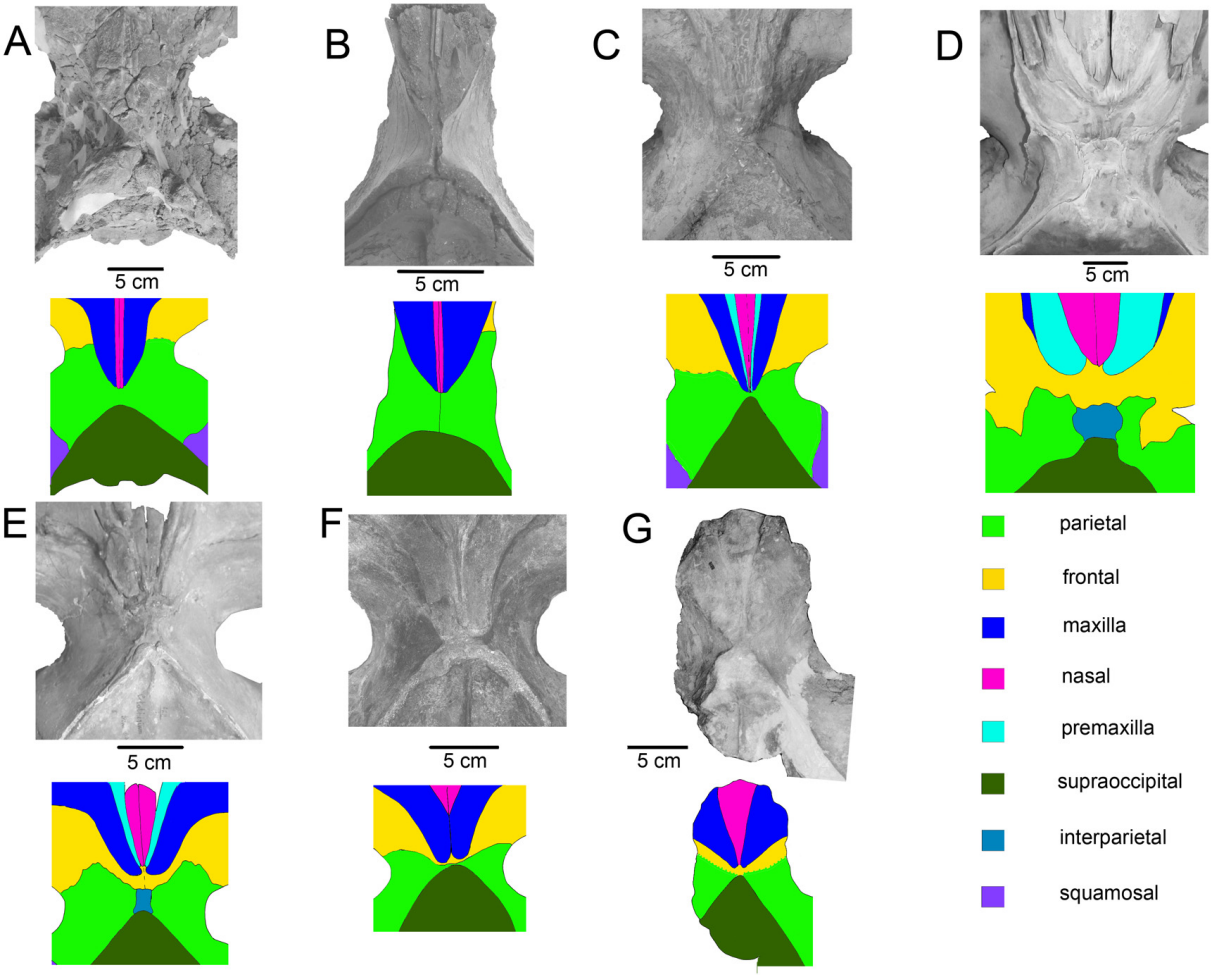
Diagnostic features of the skull vertex of Tranatocetidae and *Tranatocetus argillarius*. A, *Tranatocetus argillarius* MGUH VP 2319. B, *Mesocetus longirostris* RBINS CtM 33 / Reg. 401. C, *“Cetotherium” vandelli* UL 1. D, *Eschrichtius robustus* MVZ 125560. E, *Cetotherium riabinini* NMNH-P 668/1. F, *Piscobalaena nana* MNHN SAS 1617. G, *Metopocetus durinasus* USNM 8518. The Fig 3F is printed under a CC BY license, with permission from Felix Marx, original copyright 2012.

**Fig 4:**
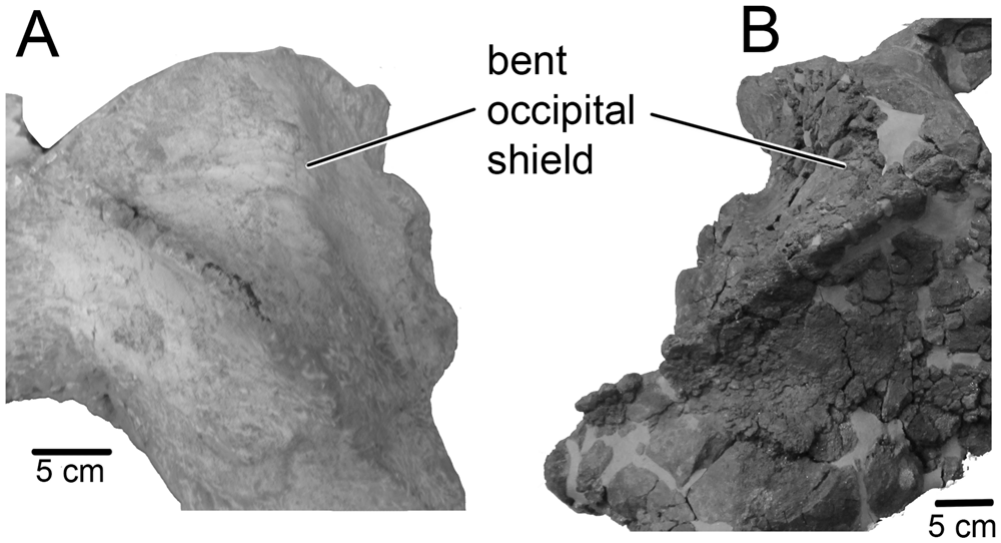
Bent occipital shield of Tranatocetidae. A, *“Cetotherium” vandelli* UL 1. B, *Tranatocetus argillarius* MGUH VP 2319.

**Fig 5:**
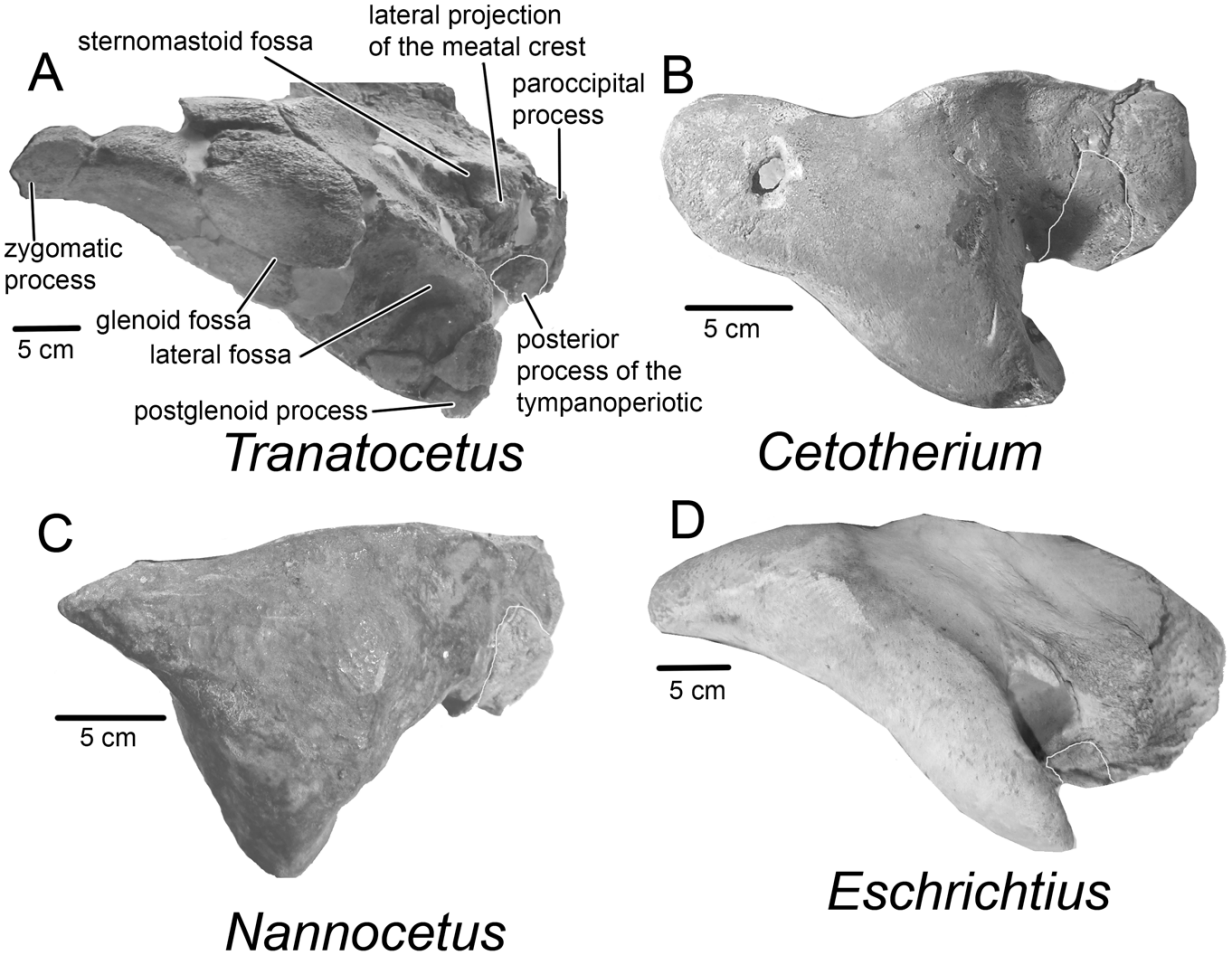
Diagnostic features of Tranatocetidae: squamosal area in lateral view. A, *Tranatocetus argillarius* MGUH VP 2319 (Tranatocetidae). B, *Nannocetus eremus* UCMP 26502 (Cetotheriidae) (inverted). C, *Cetotherium riabinini* NMNH-P 668/1 (Cetotheriidae). D, *Eschrichtius robustus* MVZ 125560 (Eschrichtiidae) (inverted). The images are cropped; the posterior process of the tympanoperiotic is delimited with a white contour.

**Fig 6:**
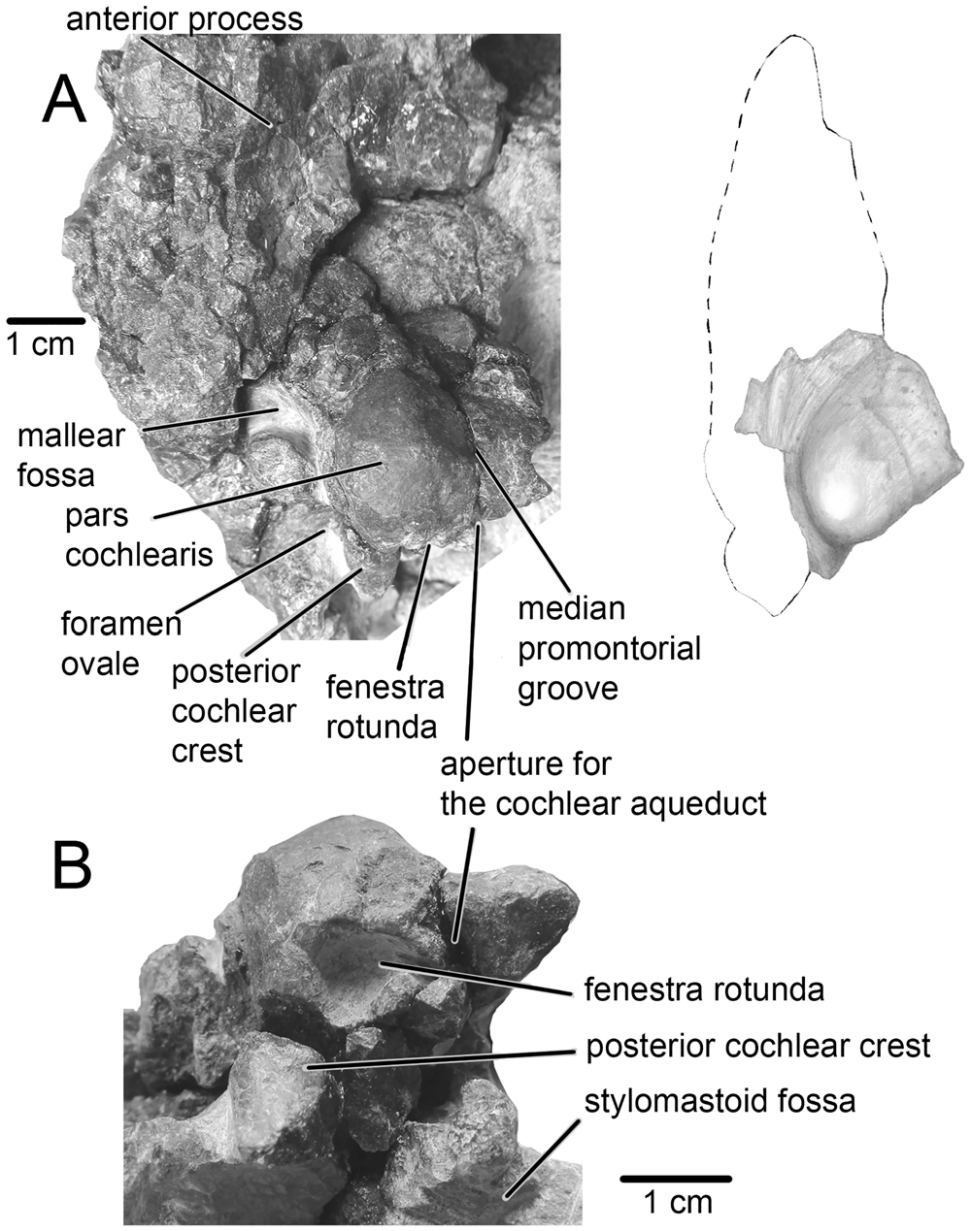
Right periotic bone of *Tranatocetus argillarius*, MGUH VP 2319. A, ventral view. B, posterior view (cropped image).

**Fig 7:**
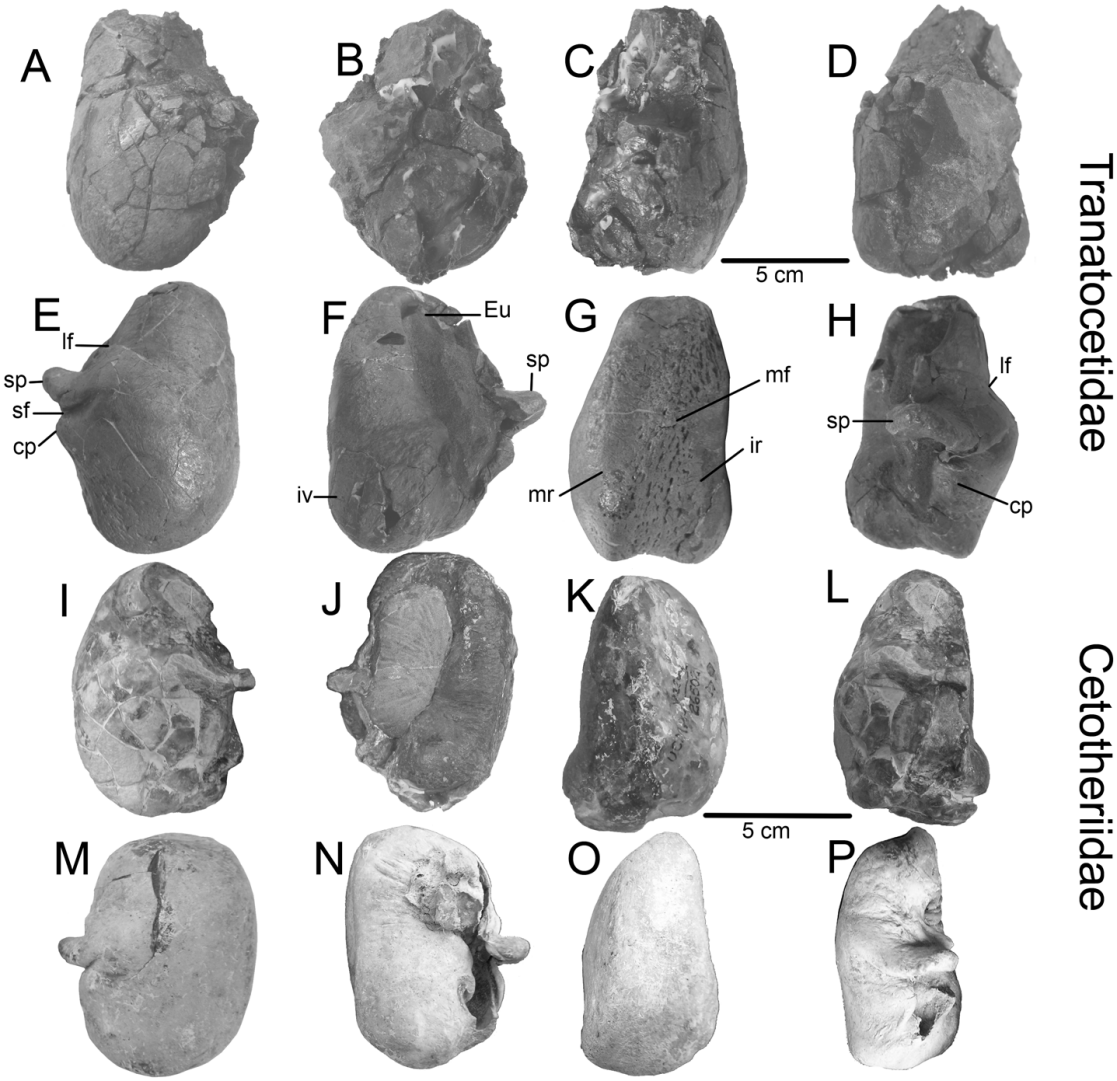
Diagnostic features of Tranatocetidae: tympanic bullae. A–D, left bulla, *Tranatocetus argillarius*, MGUH VP 2319 (Tranatocetidae). E–H, right bulla, *“Plesiocetopsis hupschii”*, RBINS 664 / Reg. 1240 (Tranatocetidae). I–L, left bulla, *Nannocetus eremus* UCMP 26502 (Cetotheriidae). M–O, right bulla, P, left bulla (inverted), *Brandtocetus chongulek* TNU Skull 4 (Cetotheriidae). A, E, I, M, ventrolateral view. B, F, J, N, dorsal view. C, G, K, O, medial view. D, H, L, P, lateral view. Abbreviations: cp, conical process; Eu, Eustachian outlet; ir, involucral ridge; iv, involucrum; lf, lateral furrow; mr, main ridge; mf, median furrow; sf, sigmoid furrow; sp, sigmoid process;

**Fig 8:**
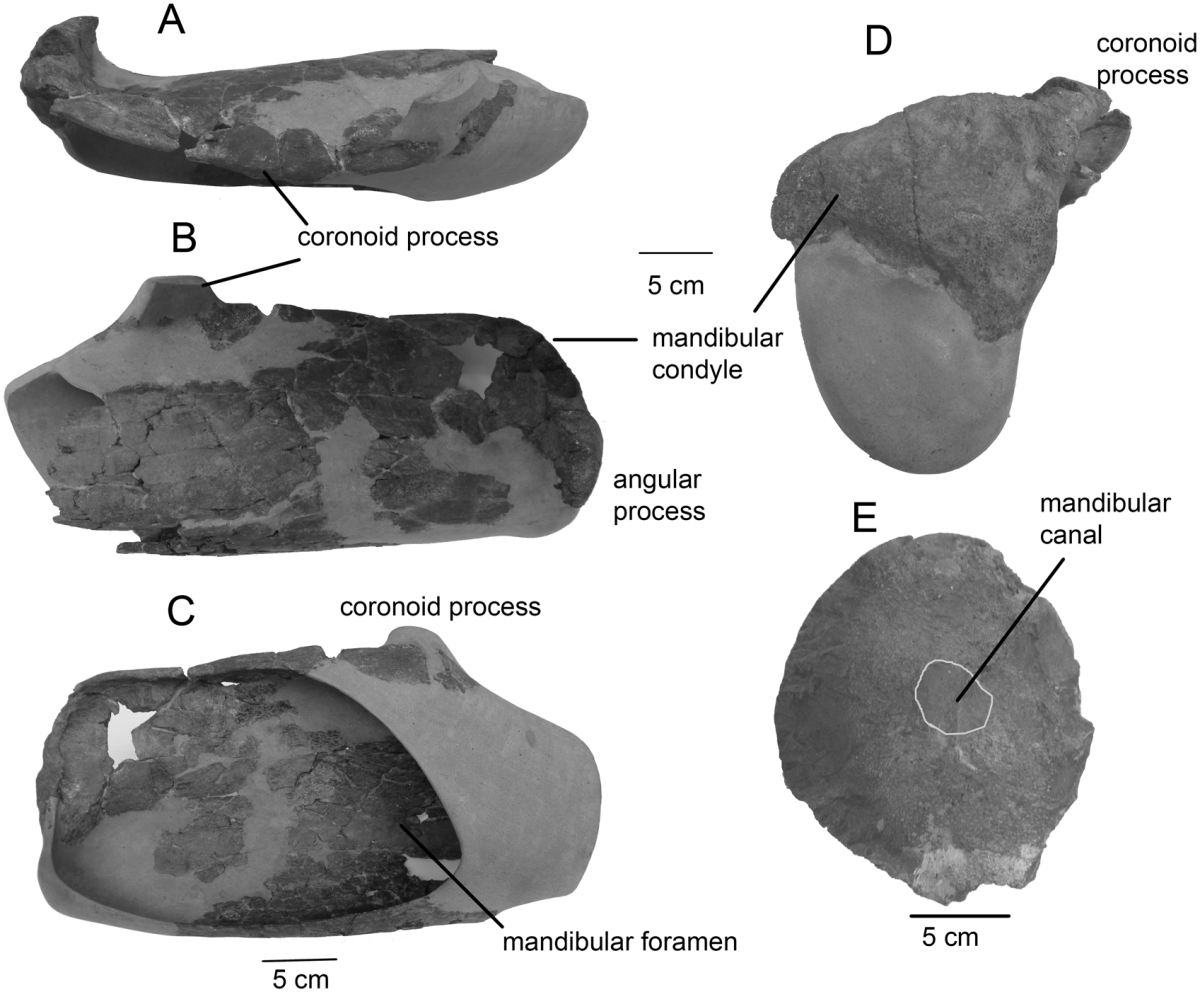
Mandible of *Tranatocetus argillarius*. MGUH VP 2319: A, dorsal view. B, lateral view. C, medial view. D, posterior view. MGUH VP 2320: F, the cross-section of the anterior portion.

**Fig 9:**
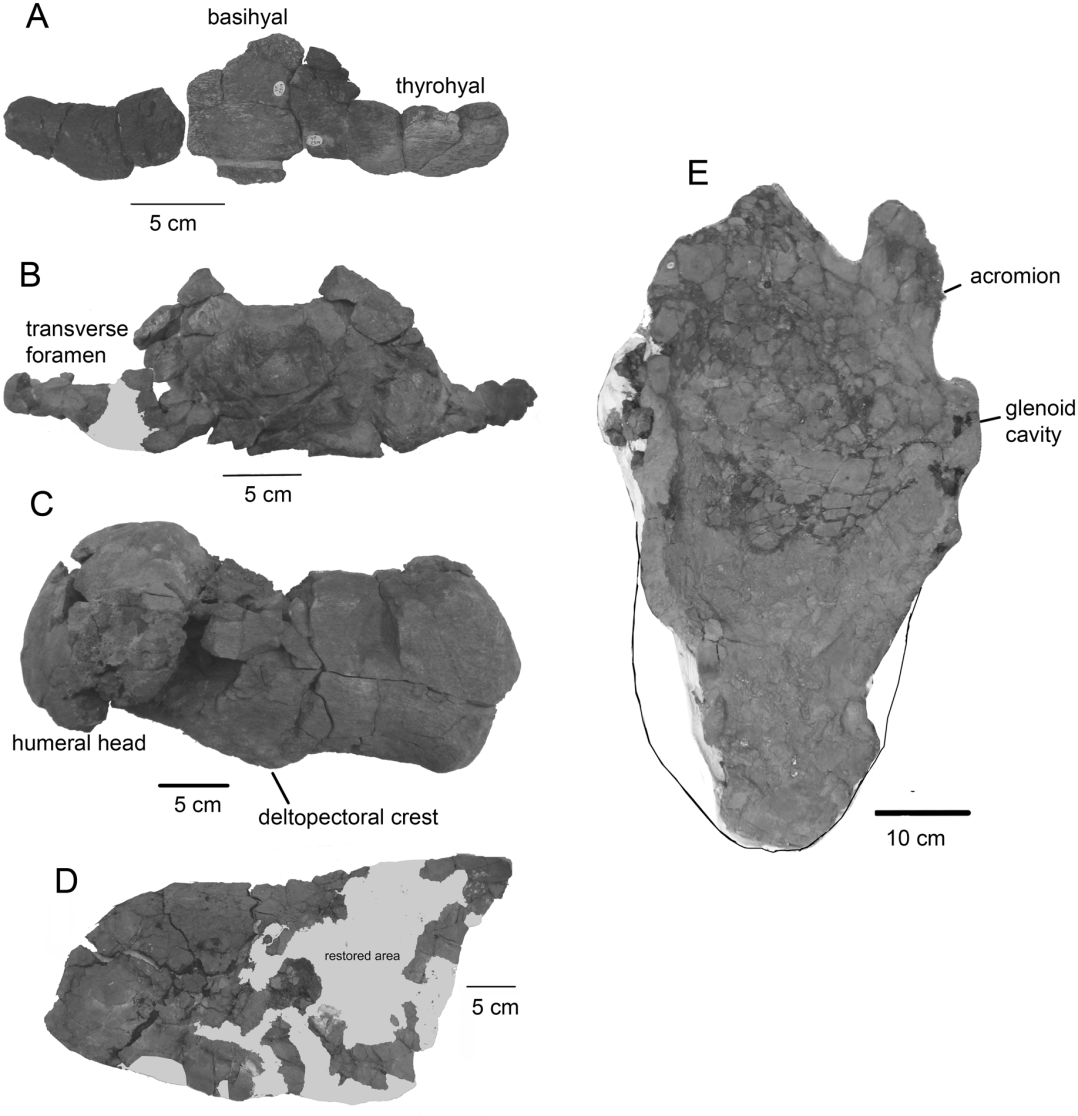
Postcranial skeletal elements of *Tranatocetus argillarius*. A–D, MGUH VP 2319: A, hyoid. B, axis. C, humerus. D, scapula. E, MGUH VP 2320, scapula in lateral view.

Two specimens of *“Mesocetus” argillarius* (MGUH VP 2319, the holotype, and MGUH VP 2320, previously undescribed specimen), housed by the University of Copenhagen, from the type locality in Gram, Denmark, were studied, including the skull and ear bones of the holotype (Figs 1–9). A phylogenetic analysis was conducted based on a data matrix of 102 unordered morphological characters (mostly adapted or modified from [5, 7, 8, 10]) and 51 taxa of cetaceans, including members of modern families Balaenopteridae, Eschrichtiidae, Neobalaenidae, Balaenidae, Cetotheriidae and closely related genera, a number of Miocene whales with uncertain affinities, the earliest toothless baleen whale *Eomysticetus whitmorei*, the basilosaurid *Dorudon atrox* and the tentative ancestor of fully aquatic cetaceans *Georgiacetus vogtlensis* (S1, S2 and S3 Appendices; S1 Table). The heuristic parsimony analysis was conducted using TNT 1.1 [13], “traditional search option”, 10000 replicates, tree bisection-reconnection branch swapping, saving 100 trees per replicate. The resulting most parsimonious trees were summarized in a strict consensus tree with zero-length branches collapsed. Branch support was estimated using symmetric resampling (change probability = 33), based on 2000 replicates and expressed as frequency differences (GC).

The anatomical nomenclature generally follows Mead and Fordyce [14]. For the abbreviations, see S1 Appendix.

### Nomenclatural acts

The electronic edition of this article conforms to the requirements of the amended International Code of Zoological Nomenclature, and hence the new names contained herein are available under that Code from the electronic edition of this article. This published work and the nomenclatural acts it contains have been registered in ZooBank, the online registration system for the ICZN. The ZooBank LSIDs (Life Science Identifiers) can be resolved and the associated information viewed through any standard web browser by appending the LSID to the prefix “http://zoobank.org/”. The LSID for this publication is: urn: lsid:zoobank.org:pub:D7CEC50A-D466-4B79-AEBD-52B7FAC85624. The electronic edition of this work was published in a journal with an ISSN, and has been archived and is available from the following digital repositories: PubMed Central, LOCKSS.

## Results

### Systematic palaeontology

**Cetacea** Brisson, 1762

**Mysticeti** Gray, 1864

**Plicogulae** Geisler, McGowen, Young and Gatesy, 2011

**Tranatocetidae, new family** *urn*:*lsid*:*zoobank*.*org*:*act*:*1FB2C6DE-7CA0-4860-BBE0-4A7C2AAD0001*

#### Diagnosis

Plicogulae sharing the following combination of specific features: (1) rostral bones override or divide frontals and contact parietals; (2) nasals (sometimes with premaxillae) divide maxillae on the vertex; (3) dorsoventrally bent occipital shield, with more horizontal anterior portion and more vertical posterior portion; (4) tympanic bulla with short, narrow anterior portion with rounded or squared anterior end and wider and higher posterior portion that is particularly swollen in the posteroventral area; a moderately deep lateral furrow and a shallow groove posterior to the sigmoid process; well-developed conical process.

Differing from Cetotheriidae and related taxa in: having a deep anteriorly, anteroventrally or anterolaterally (rather than anteromedially) oriented glenoid fossa; the posterior process of the tympanoperiotic slightly (rather than broadly) exposed on the posterolateral wall of the skull; short angular process of the mandible and the broad and low condyle oriented in the same plane as the ramus. Differing from *Pinocetus*, *Cephalotropis* and Neobalaenidae in the shape of tympanic bulla: the inflated posterolateral (rather than anterolateral) end and a shallower lateral furrow. Differing from *Titanocetus*, Balaenopteridae and Eschrichtiidae in: the absence of the anterior wing of the parietal bone (present in *Mixocetus*); the triangular occipital shield with the sharp anterior end (rounded in *Mixocetus*); the long coronoid process and dorsally open sub-condylar furrow of the mandible. Differing further from Balaenopteridae and Eschrichtiidae in the thin and long zygomatic process of squamosal; the paroccipital process protruding far posterior to the postglenoid process; the anterior margin of the supraoccipital posterior to the centre of the temporal fossa; the distally broadening posterior process of the tympanoperiotic exposed on the posterolateral wall of the skull. Sharing with Cetotheriidae, Balaenopteridae and Eschrichtiidae the X-shaped skull vertex formed by approximating or contacting occipital shield and maxillary elements (combined telescoping of skull); squamosal cleft (except in some cetotheriids); well-developed anterior process of the periotic that is at least as long as its lateral flange (or lateral tuberosity).

#### Included taxa

*“Aulocetus” latus*, *“Cetotherium” megalophysum*, *“Cetotherium” vandelli*, *Mesocetus longirostris*, *Mixocetus elysius*, *“Plesiocetopsis hupschii”* sensu Van Beneden, 1886, *Tranatocetus argillarius*

#### *Tranatocetus*, gen. nov

urn:lsid:zoobank.org:act:53F4A70B-5F67-4D8D-AFAC-FD308974D066

#### Type and only known species

*Tranatocetus argillarius* (Roth, 1978)

#### Diagnosis

As for species.

#### Etymology

*Tranato* (latin) swimming across, *cetus* (from greek *ketos*) sea monster/whale.

### *Tranatocetus argillarius* (Roth, 1978), comb. nov

Synonymy: *Mesocetus argillarius* Roth, 1978; *‘Mesocetus’ argillarius* Steeman, 2007

#### Holotype

Geological Museum of the University of Copenhagen (MGUH VP 2319) consisting of partial skull with periotics and left tympanic bulla but lacking rostrum; the proximal portion of the lower jaw; partial hyoid bone (basihyal and thyrohyals); thirty-three vertebrae; ribs and rib fragments; parts of both scapulae; humerus and two phalangeal bones.

#### Referred specimens

MGUH VP 2320 consisting of six mandible fragments (anterior portion), seven thoracic and seven lumbar vertebrae and both scapulae, one of which is sub-complete.

#### Locality and age

Gram clay pit, 1.5 km north of the town of Gram, South Jutland, Denmark (geographic coordinates 55°18' N, 09°04' E). Gram Formation, Late Miocene [15]: based on the composition of associated mollusc fauna, the specimen can be accurately identified as belonging to layer 3 or 4 of the Gram Clay Formation [15,16] which are dated to mid-Tortonian, ca. 9.9–8 mya according to a combined study of geomagnetic variation, dinoflagellates and malacofauna [16–18].

#### Diagnosis

Modified from [12]. The scapula is anteroposteriorly elongated; the glenoid cavity displaced toward the anterior end. The deltopectoral crest of the humerus is long. Differing from all Plicogulae, except *Mesocetus longirostris*, *Pelocetus calvertensis* and *Parietobalaena campiniana*, in: retaining plesiomorphic features of the lower jaw: the dorsal border in front of the mandibular condyle gradually ascending towards the coronoid process, wide mandibular foramen and wide mandibular canal. Differing from *Mesocetus longirostris*, *“Aulocetus” latus*, “*Cetotherium*” *megalophysum* and “*Cetotherium*” *vandelli* in: the wide skull with laterally expanded squamosals; straight ascending processes of maxillae which extend parallel to each other (rather than tapering and converging posteriorly); small lateral projection of the posterior meatal crest on the posterolateral side of the postglenoid process and paroccipital processes extending far posterior to the occipital condyles. Sharing only with *Mesocetus longirostris*: posterior ends of premaxillae fused with the maxillae and divided on the vertex by long, narrow and high (vertical plate-like) nasals; cervical vertebrae with wide transverse foramina, almost as wide as the centra (see also [12]).

### Description

Of the **skull,** a distinctly short, wide and high neurocranium with posterior portions of the rostral bones has been preserved (Figs 1 and 2; Table 1). In lateral view, the vertex forms an angle close to 90° between the facial bones and the occipital shield. The temporal crest is high, so that the vertex is highly elevated above the rhomboid temporal fossa. Viewed anteriorly, the skull is slightly asymmetrical as the vertex is shifted left, and the occiput is smaller to the left relative to the right: this seems to be the natural condition. The posterior portion of the **premaxilla** is not distinct and may be fused with the maxilla (Fig 3). The ascending process of the **maxilla** is posteriorly constricted. The ascending rostral processes completely override the frontals on the vertex and overlap the parietals. The anterior edge of the maxilla is strongly concave at the base of the ascending process leaving a crescent-shaped portion of the supraoccipital process of the frontal anterior to a well-defined transverse orbitotemporal crest. This may mark the insertion of the occipitofrontalis muscle [19]. The straight, long, narrow and high **nasals** are wedged between the ascending processes of the maxillae, dividing these on the vertex to their posteriormost extension. The supraorbital process of the **frontal** gradually slopes ventrally. The lateral portion of the **parietal** is concave. The parietals contact in a short and low sagittal crest on the vertex. The **occipital** shield is sub-triangular, with high, sigmoid nuchal crests. A low horizontal crest over the dorsal condyloid fossa divides the occipital shield into a nearly vertical posteroventral portion that meets a more horizontal anterodorsal portion at a blunt angle (Fig 4). The long and robust paroccipital process extends posterior to the condyle and postglenoid process.

**Table 1:** Cranial measurements (mm) of *Tranatocetus argillarius* MGUH VP 2319.

Measurement	Distance
Bizygomatic width	814
Length of the neurocranium	520e
Nasal length	200e
Minimum intertemporal width	217
Greatest length of temporal fossa	257
Greatest width of temporal fossa	260
Distance between tip of zygomatic process and tip of postglenoid process	300
Width between paroccipital processes	468
Median length of occipital shield	271
Width of foramen magnum	61
Height of foramen magnum	69
Bicondylar width	172
Condylar height	103
Width between foramina pseudovale	292
Width between lateral margins of basioccipital crests	195
Width between posterior-most points of postglenoid processes	667
Greatest height of neurocranium	302
Length of pars cochlearis	18
Height of pars cochlearis	17
Length of posterior process of tympanoperiotic	135
Length of tympanic bulla	85
Maximum width of tympanic bulla	65
Posterior width of tympanic bulla in medial view	50
Posterior height of mandible	160e

The **squamosal** is wide in both medial and lateral portions. The squama bulges into the temporal fossa and a squamosal cleft is present. The lateral portion with the zygomatic and postglenoid process is transversely expanded, and situated entirely lateral to the paroccipital process. The anteriorly directed, thin finger-like zygomatic process is slightly laterally curved. The squamosal prominence is well developed dorsally, forming a high and narrow crest. In lateral view, the postglenoid process is low and ventrally directed, anterodorsally thick and robust and has a round bulge posterodorsally. In posterior view, it is transversely wide. The glenoid fossa faces ventrally to anteroventrally. There is a well-developed fossa on the lateral side of the squamosal (named here as the lateral fossa), which joins the glenoid fossa: it is surrounded dorsally by a prominent crest on the zygomatic and postglenoid process. The sternomastoid fossa is deep and the lateral projection of the meatal crest delimiting it ventrally is distinct but small (Fig 5). The falciform process bulges ventrally. Ventrally, the skull surface is weathered and the sutures between the bones are mostly obliterated, and much of the bone outlines can be only tentatively reconstructed. The **palatine** has convex (rather than notched) anterior and posterior margins. The **pterygoid** contacts the squamosal and is almost completely covered by the palatine. The pterygoid sinus is transversely wide. The **vomer** extends posteriorly to the **basioccipital** crest. This crest is bulbous, anteroposteriorly elongated and as large as the tympanic bulla. The fossa for the tympanohyal ligament is well developed, and it is surrounded with a high crest.

The **periotic** body is significantly larger than the pars cochlearis (Fig 6). The pars cochlearis is small, anteroposteriorly short and bulges slightly ventral to fenestra rotunda. The anterior process of the periotic is sub-triangular with a poorly developed lateral flange (or lateral tuberosity). The mallear fossa is small, round and shallow. The posterior cochlear crest (caudal tympanic process) is short and the stylomastoid fossa is shallow. The groove for the tensor tympani muscle is shallow. The posterolaterally directed posterior process of the tympanoperiotic is long, widening distally with a small exposure on the posterolateral wall of the skull. The **tympanic bulla** is pyriform in ventral view (Fig 7). The narrow and low anterior portion is short with a rounded anterior end, while the posterior portion is larger in all dimensions. Particularly swollen is the posteroventral area lateral to the base of the posterolaterally directed sigmoid process. The lateral furrow is deep and the groove posterior to the sigmoid process is shallow (see also [5]). In ventrolateral view, the conical process, as preserved, is distinctly high. In medial view, the main ridge is more developed than the involucral ridge. The ridges converge toward the anterior end. The lateral lobe extends more posteriorly than the medial one. In dorsal view, the involucrum is high and convex.

The **mandible** is plesiomorphic in having a high mandibular foramen and opening for the mandibular canal, a wide and low condyle, and the dorsal border gradually ascending from the condyle to the coronoid process. The mandibular canal preserved in MGUH VP 2320 strongly narrows anteriorly (Fig 8). For detailed description, see [12]. The preserved portion of the **hyoid** apparatus comprises the basihyal and the thyrohyals, all of which are dorsoventrally flattened (Fig 9A).

The presumed vertebral formula is C_7_T_13_L_12_Ca_3+_ (7 cervicals, 13 thoracics, 12 lumbars and at least 3 caudals). All **vertebrae** (including all cervicals) are unfused. All epiphyses in MGUH VP 2319 are fused with the centra, indicating full physical maturity. The cervical vertebrae have large transverse foramina, which are sometimes as wide as the centra, and even the axis has wide and high foramina (Fig 9B). The thoracic and lumbar vertebrae gradually elongate and become higher towards the tail. The longest are the four posteriormost lumbars and the caudals, all of which are longer anteroposteriorly than they are wide transversely.

The **scapula**, well preserved in MGUH VP 2320 and fragmentary in MGUH VP 2319, is very long (Fig 9D and 9E). The anteroposterior length is 2.5 times greater than the height at the glenoid cavity due to elongation of the posterior portion. The glenoid cavity is located relatively far anteriorly. The acromion is high and long, whereas the coracoid process is short. The **humerus** is narrow, with a low but distinct and long deltopectoral crest. The compact head is nearly vertical, and it takes one third the length of the bone (Fig 9C).

### Phylogeny

**Fig 10:**
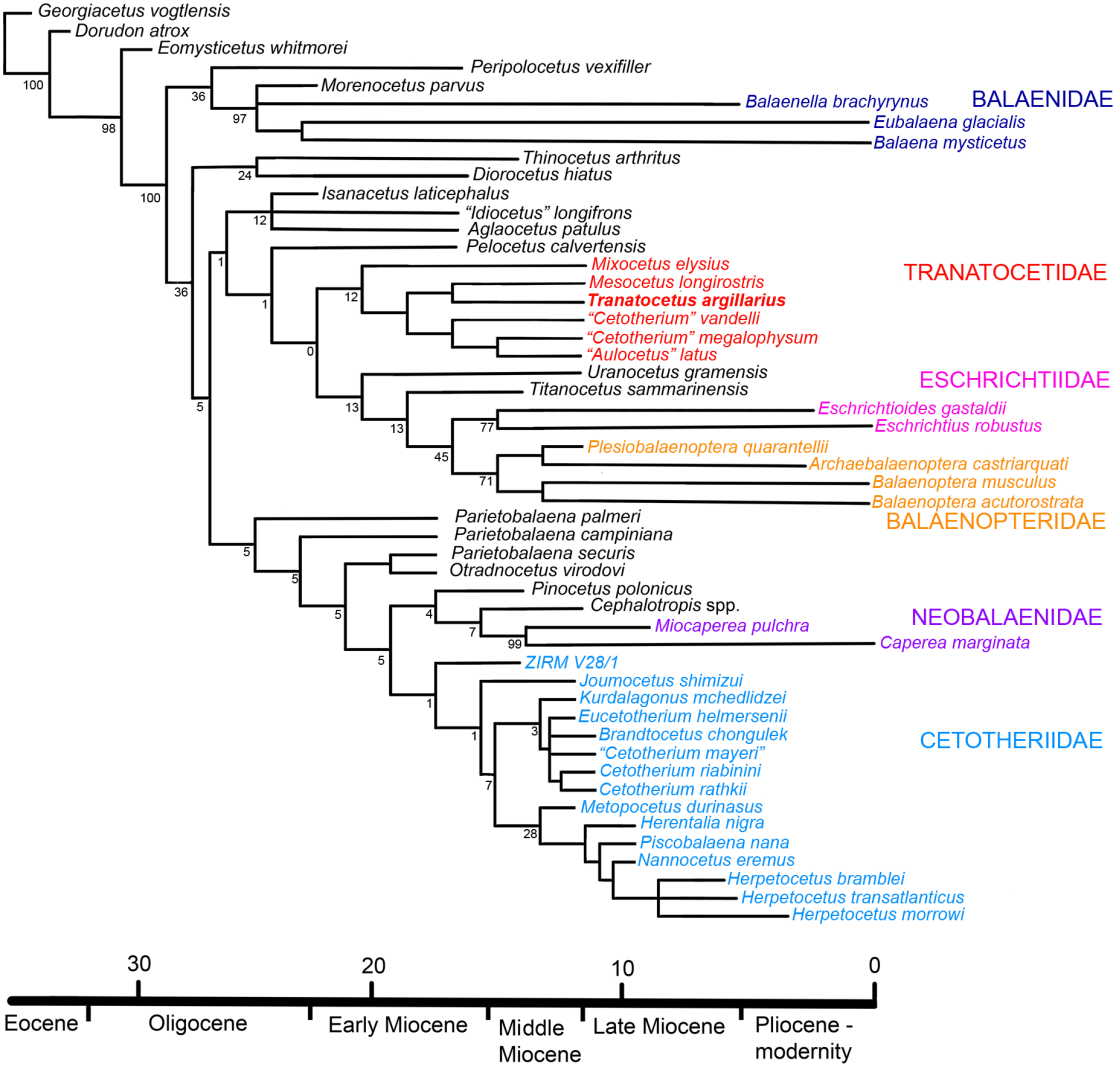
The phylogenetic tree of *Tranatocetus argillarius* and related taxa of baleen whales. The tree is the consensus of 12 most parsimonious trees (501 step, CI = 0.33, RI = 0.66). The age values are provisionally indicated as the earliest estimates and are based on the review by Fordyce and Marx [8], as well as on original descriptions.

The phylogenetic analysis supports a monophyletic Plicogulae comprising Tranatocetidae, Balaenopteridae, Eschrichtiidae, Cetotheriidae and Neobalaenidae, as well as a number of mid-Miocene whales (Fig 10). Plicogulae split into two major branches, one containing Cetotheriidae and Neobalaenidae, the other Tranatocetidae, Balaenopteridae and Eschrichtiidae. The basalmost described cetotheriid is *Joumocetus shimizui* [20]. The clade including the recent pygmy right whales (Neobalaenidae) also includes the Late Miocene genus *Cephalotropis* and has *Pinocetus polonicus* from the Middle Miocene of Poland [21] as the basalmost member. The stem taxa for both Cetotheriidae and Neobalaenidae are the Middle Miocene taxa *Otradnocetus* and *Parietobalaena*, the latter of which appears as a paraphyletic group. Thus, Cetotheriidae and Neobalaenidae, as well as *Cephalotropis*, *Pinocetus*, *Otradnocetus* and *Parietobalaena*, are classified here as superfamily Cetotherioidea [5]. Other mid-Miocene non-balaenid taxa included in this analysis are classified as stem Plicogulae (Diorocetidae) or stem members of the Balaenopteridae clade. Balaenopteridae and Eschrichtiidae form a clade with *Titanocetus* [22] and *Uranocetus* [23]. Tranatocetidae is the sister group to this clade. *Tranatocetus argillarius* and *Mesocetus longirostris* [24, 25] are pooled together in the cladogram based on the common primitive traits: anteroventrally directed glenoid fossa, sigmoidal nuchal crests and the large mandibular foramen. Tranatocetidae also includes *“Aulocetus” latus*, “*Cetotherium*” *megalophysum*, “*Cetotherium*” (or *“Metopocetus”*) *vandelli* and *Mixocetus elysius*. Another related taxon, not included in the analysis, is “*Plesiocetopsis hupschii*”, as identified by Van Beneden [25] (Fig 7E–7H) which is represented by the isolated fragments: it is unclear if it relates to the holotype of this species [26, 27] which is currently unavailable for study [28]; in its turn, this holotype, as illustrated by [27], is strongly similar to *Mesocetus longirostris* [25]. All of these taxa have been mentioned and described as representatives of different families and genera [5, 7, 10, 28–33], and their anatomy, taxonomy and relationships need further revision.

## Discussion and Conclusions

Previous attempts to unravel mysticete phylogeny have been plagued by numerous controversies, and several groups seem to have conflicting combinations of character traits. This was observed well before phylogenetic studies became common, by Kellogg [34] in his description of *Mixocetus*, as is also evident from the name he gave this whale. Members of the new family, Tranatocetidae, display a combination of characters often associated with either Balaenopteridae (rorquals) or Cetotheriidae (cetotheres), as can be seen on the illustrations of a few tranatocetid taxa by Kellogg [35] (see figures 389, 420–421, 449, 459–460, 470, 542–543, 548–553 in [35]). Rorqual-like features include a deep anteroventrally to anterolaterally oriented glenoid fossa (Fig 5), a short angular process of the mandible and a broad and low mandibular condyle (Fig 8). Cetothere-like features are the absence of the anterior wing of the parietal bone and the triangular occipital shield with a sharp anterior end (both traits are not shared with *Mixocetus*), the anterior margin of the supraoccipital posterior to the centre of the temporal fossa, the paroccipital process protruding far posterior to the postglenoid process, and the distally broadening posterior process of the tympanoperiotic (Fig 1).

Tranatocetidae show a unique type of the telescoping of the rostral bones where the premaxillae and nasals divide the maxillae on the vertex, and the rostral bones are wedging far posterior on the skull roof, overriding the frontals and interdigitating with the parietals (Fig 3). This anatomy is superficially similar to that of Cetotheriidae and some tranatocetids were previously considered to be cetotheres and even identified as *Cetotherium*. The most recent studies placed “*Cetotherium*” *megalophysum* as a closely related taxon to *Nannocetus* [10] or *Metopocetus* and *Piscobalaena* [11, 36] all of which at some point were pooled together with *Herpetocetus* within Cetotheriidae [5, 11]. Indeed, tranatocetids and cetotheriids share the advanced telescoping of the rostral bones (Fig 3). Moreover, the premaxillae are not dorsally exposed in the latter three cetotheriid genera [10], as well as in *Tranatocetus*, but contrary to *Cetotherium* [37]. However, the medial portions of cetotheriid frontals are thrust backwards together with the rostral bones, and they are usually exposed on the vertex posterior to the rostral bones (Fig 3F and 3G). Only in some specimens of *Piscobalaena* (Fig 3F), the frontoparietal suture seems to be overridden by the maxillae, but this suture is oblique and is directed anterolaterally, as viewed from the dorsal side, which is the typical cetotheriid frontal anatomy [4]. The ascending processes of the maxillae in such cetotheriids, as *Herpetocetus* and *Piscobalaena*, roof the nasals and posterior ends of the premaxillae, as well as the medial portions of the frontals, from the dorsal side [4, 10]. Also in *Herpetocetus* and related taxa (but not in *Cetotherium*) the premaxillae end well anterior to the maxillae and nasals [10]. The only taxon in which this anatomy is unclear is *Metopocetus durinasus*, known only by the holotype. This is due to the obliteration of cranial sutures. *Metopocetus* still has the ascending processes of the maxillae approximating each other at their posteriormost extend, which is typical for cetotheriids [37] (Fig 3F). Contrary to any cetotheriid state, Tranatocetidae usually have transversely oriented frontoparietal sutures that are always clearly situated anterior to the ends of the rostral bones [35] (Fig 3A–3C). The ascending processes of the maxillae neither contact with each other nor roof the medially lying bones [35]. Instead, they often lie in parallel to each other and, if converging, constrict or even squeeze the premaxillae. They may fuse with the premaxillae and also fuse and anastomose with the underlying frontals, as is the case in *Mesocetus longirostris* RBINS CtM 33 / Reg. 401, which was illustrated by Van Beneden [25] (see pl. 34, Figure 3 in [25]).

A diagnostic trait of Cetotheriidae, which is shared by all the included taxa, is a shallow glenoid fossa [37] (Fig 5B and 5C). *Tranatocetus*, as well as all other Tranatocetidae [35], has quite a different anatomy (Fig 5A). Notably, it has been already mentioned that *Mixocetus* did not match the diagnosis of Cetotheriidae in this aspect [37]. The unique anatomy of the tympanic bulla of Tranatocetidae, with the short anterior portion, swollen posterior portion and wide and high conical process, strikingly differs from the oval or box-shaped bulla with a small conical process, which is typical for all cetotheriids and related taxa (Fig 7). Unfortunately the bulla is missing in the holotype of *Metopocetus durinasus*, but it can be compared with *Tranatocetus* based on its periotic bone. In *Tranatocetus* the pars cochlearis bulges ventrally to the fenestra rotunda, and it is dorsomedially receding in posterior view, whereas these traits are lacking in *Metopocetus*, as well as in the other cetotheriids.

Finally, the mandible anatomy of Cetotheriidae is characterized by very distinct synapomorphies: “an angular process extending posterior to the condyle, a condyle oriented obliquely to the long axis of the body, and a small and laterally bent coronoid process” [37]. The position of the angular process and the condyle has been considered as the only unambiguous synapomorphy of Cetotheriidae [10]. *Tranatocetus* and *Mixocetus*, the tranatocetids with the mandibles preserved, do not share this anatomy [34] (Fig 8) and thus do not match the definition of Cetotheriidae.

The postcranial skeleton of *Tranatocetus* shows the unique apomorphies among all baleen whales. The hyoid is particularly small and flat. It is only slightly larger than in *Cetotherium riabinini*, an extremely small whale with a 326 mm wide skull [37], and significantly smaller than in any extant species. Unlike all mysticetes (including Balaenidae, Balaenopteridae, Cetotheriidae, Eschrichtiidae and Neobalaenidae) but similar to many odontocetes, the thyrohyals are dorsoventrally flat (rather than thick and round or oval on the cross-section) and form a flat (rather than obtuse) angle. However, unlike odontocetes or balaenids and eschrichtiids, but similar to balaenopterids and cetotheriids, the thyrohyals are posteriorly bowed and laterally (rather than posterolaterally) directed. The shape of scapula in *Tranatocetus argillarius* is highly derived among all mysticetes. The proportions are similar to the scapula of *Piscobalaena nana* [4], the anterior shift of the glenoid cavity, and the anatomy of acromion are found in *Pinocetus polonicus*, but the combination is unique. Also, the unusual anatomy of humerus is more similar to that of physeteroids than to that of any baleen whales.

The result of this phylogenetic analysis (including several problem taxa) shares elements found in previous studies [3, 5, 7–10, 20, 37, 38]. However, this novel combination of taxa and characters leads us to identify a new family, Tranatocetidae, which may be closer related to Eschrichtiidae and Balaenopteridae than to Cetotheriidae. *Parietobalaena* and *Otradnocetus* are the basalmost cetotherioids: this similarity is based on the periotic morphology and, in the case of *Otradnocetus*, on the anatomy of the posterior portion of the mandible [39]. Neobalaenidae are closely related to *Cephalotropis* and *Pinocetus*. Thus, we obtained a taxonomic structure of Plicogulae with the five families Balaenopteridae, Eschrichtiidae, Tranatocetidae, Neobalaenidae and Cetotheriidae, and a number of basal taxa. This interpretation is well supported by molecular data [6, 8] (and references therein), and by the chronology of the fossil record, as crown taxa are mostly preceded by the basal groups. The minimum age of Plicogulae in this analysis is marked by the occurrence of *Isanacetus laticephalus* by Burdigalian, 17.5–16 mya; however, it can be extended as early as 25–23 mya if the Late Oligocene *Mauicetus parki* belongs to this group too [5, 36].

**Fig 11:**
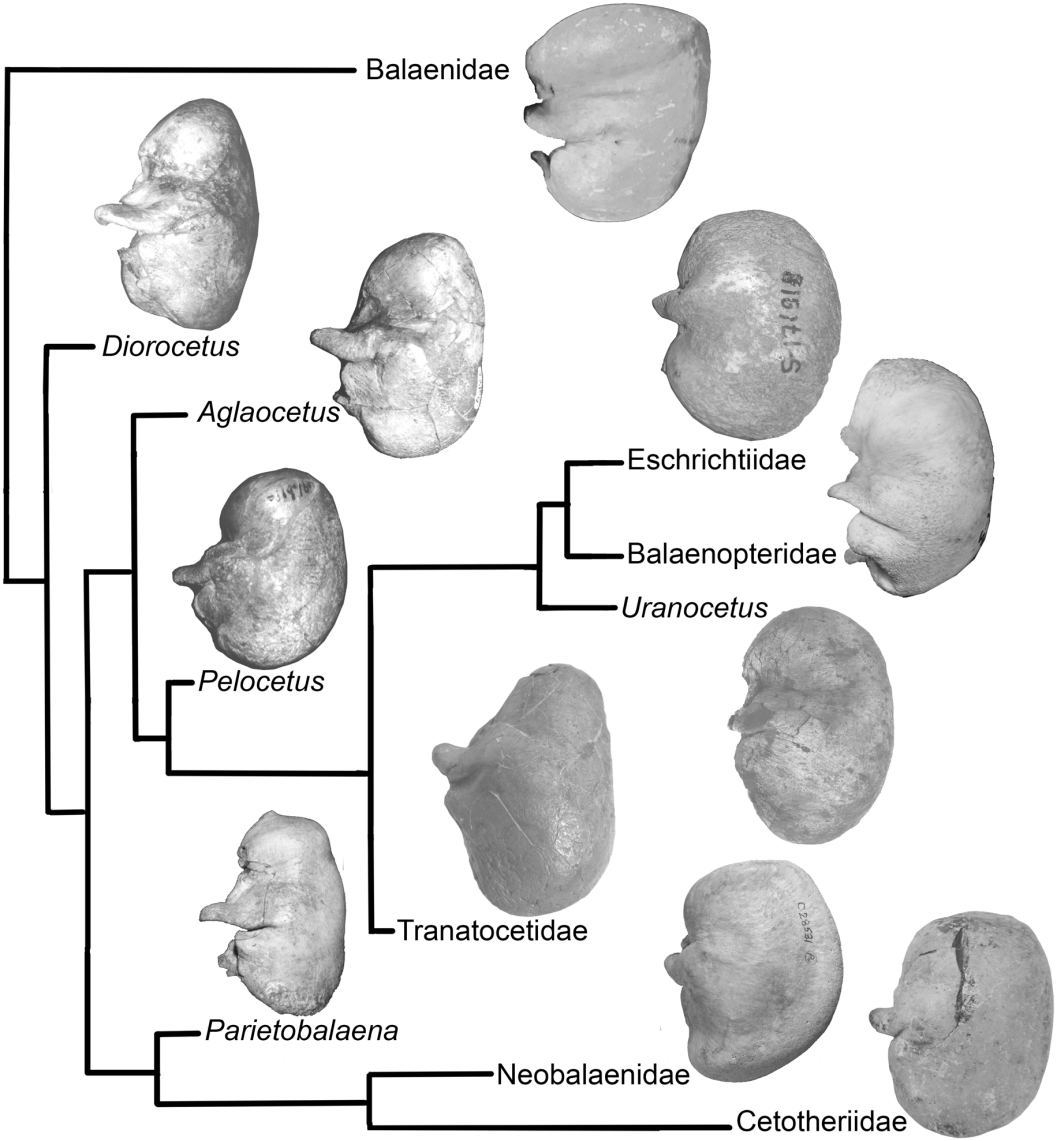
The shape of the tympanic bulla (ventrolateral view) in the phylogeny of baleen whale families. Balaenidae: *Eubalaena glacialis*, CU CN 1x. *Diorocetus*: *Diorocetus hiatus*, USNM 23494. *Aglaocetus*: *Aglaocetus patulus*, USNM 23690. Eschrichtiidae: *Eschrichtius robustus*, ZMMU 171918. *Pelocetus*: *Pelocetus calvertensis*, USNM 11976. Balaenopteridae: *Balaenoptera acutorostrata*, ZMMU 171919. Tranatocetidae: *“Plesiocetopsis hupschii”*, RBINS 664 / Reg. 1240. *Uranocetus*: *Uranocetus gramensis*, MSM P813. *Parietobalaena*: *Parietobalaena palmeri*, USNM 16119. Neobalaenidae: *Caperea marginata*, NMV C28531; printed under a CC BY license, with permission from Felix Marx, original copyright 2012. Cetotheriidae: *Brandtocetus chongulek*, TNU Skull 4.

A unique shape of the tympanic bulla can identify members of Tranatocetidae. Noticeably, this analysis shows that all other Neogene and modern mysticete families also have unique bulla shapes indicating a possible self-sufficient diagnostic feature of family-level groupings (Fig 11). The common basal morphological type of the bulla in Plicogulae is found in *Diorocetus*: it is characterized by a long anterior portion, a long (transversely wide) sigmoid process with a swollen area near its base, a deep lateral furrow and a deep groove posterior to the sigmoid process, a low conical process and a posteriorly deep median furrow. This morphology is largely retained in *Parietobalaena palmeri*, except for the latter trait. Crownwards in this lineage, in Neobalaenidae and Cetotheriidae the sigmoid process is short and the groove posterior to the sigmoid process is shallow. In Neobalaenidae the anterior portion is low with the swollen anterolateral area, and in Cetotheriidae the lateral furrow is shallow and the base of the sigmoid process is not swollen. In another branch, *Aglaocetus patulus* shares the swollen anterolateral area with Neobalaenidae, whereas *Pelocetus* gains a high conical process, and the crownward taxa of this lineage have a short sigmoid process. Tranatocetidae have a short anterior portion, a shallower (although still well developed) median furrow and a high conical process, as well as the swollen posterior portion. The deeper lateral furrow and the higher (although not as high as in *Pelocetus*) conical process distinguish Eschrichtiidae from Balaenopteridae, whereas the latter family is distinct in having a large anterolateral shelf.

The possibility of identifying mysticete families based on bulla anatomy is particularly important in applied systematics and evolutionary studies of baleen whales because mysticete evolution is generally characterized by numerous homoplasies. For example, the X-shaped skull vertex is shared by balaenopterids and cetotheriids, and an arched rostrum is found in *Isanacetus*, *Eschrichtius*, Neobalaenidae and Balaenidae (in which it reaches its most extreme manifestation). The cetacean hearing apparatus is more conservative [40], and its skeletal parts are promising for describing the cetacean systematics and phylogeny [5, 41]. However, the first attempt to classify mysticetes based on the earbones [5] was not supported by other studies [7, 9, 10] except for the most controversial phylogenies nesting Neobalaenidae within Cetotheriidae [8, 36]. Broader taxonomic sampling, including the previously described European taxa with uncertain affinities (also reviewed in: [5, 28]), results in a phylogeny and classification that partly reconciles the most recent studies [3, 8–10, 36] and reveals hitherto hidden lineages.

Inclusion of problem taxa such as *Tranatocetus argillarius* in phylogenetic studies brings new light to the understanding of the character distribution and the diagnostic value of character traits. For example, the retention of a large mandibular foramen is known to be a plesiomorphic trait, which is present in *Tranatocetus argillarius* as well as in *Diorocetus hiatus*, other mid-Miocene whales, and eomysticetids. The presence of a bulge ventral to the fenestra rotunda in the pars cochlearis of the periotic is likewise shared by *Tranatocetus argillarius*, *Diorocetus hiatus*, and *Uranocetus gramensis*, indicating that this is a retained plesiomorphic trait, rather than a shared apomorphy supporting a close relationship between the latter taxa, as indicated by a previous study [23]. This highlights the importance of careful taxa selection, as well as the need for revisiting previously partially described specimens and review them in the light of the wealth of new information published in later years.

## Supporting Information

S1 Appendix Institutional abbreviations. (DOC)

S2 Appendix List of the specimens used in the analyses. (DOC)

S3 Appendix Characters used in phylogenetic analysis. (DOC)

S1 File Permission from the copyright holder. (PDF)

S1 Table Character-taxon matrix used for phylogenetic analysis. (DOC)
