# Supplementary material for: From Problem Taxa to Problem Solver: A New Miocene Family, Tranatocetidae, Brings Perspective on Baleen Whale Evolution
Source: PLoS One. 2015 Sep 2;10(9):e0135500. doi: 10.1371/journal.pone.0135500 (PMC4558012; doi:10.1371/journal.pone.0135500)
Supplement: S1 Appendix — (DOC) [file pone.0135500.s001.doc]

**S1 Appendix Institutional abbreviations**

CAS, Californian Academy of Sciences, San Francisco CA, USA

CMT, Central Museum of Taurida, Simferopol, Ukraine

CU, University of Copenhagen, Denmark

GMNH, Gunma Museum of Natural History, Gunma, Japan

GSM, Georgia Southern Museum, Statesboro GA, USA

MFM, Mizunami Fossil Museum, Gifu, Japan

MGGC, Museo Geologico Giovanni Capellini, University of Bologna, Italy

MGPT, Museo Regionale di Scienze Naturali di Torino, Turin, Italy

MGUH VP, Museum Geologicum Universitatis Hauniensis (Vertebrate Palaeontology), Geological Museum of the University of Copenhagen, Denmark

MLP, Museo de Ciencias Naturales de La Plata, La Plata, Argentina

MPST, Museo Paleontologico “Il Mare Antico”, Salsomaggiore Terme, Italy

MSM, Department of Natural History and Palaeontology, Museum of Southern Jutland, Gram, Denmark

MVZ, Museum of Vertebrate Zoology, University of California, Berkeley CA, USA

MZ, Museum of the Earth, Polish Academy of Sciences, Warsaw, Poland

NMB, Natuurmuseum Brabant, Tilburg, Netherlands

NMG, National Museum of Republic of Georgia, Tbilisi, Georgia

NMNH-P, Paleontological Museum of the National Museum of Natural History, Kiev, Ukraine

NMRA, National Museum of the Republic of Adygeya, Maikop, Russia

NMV, Museum Victoria, Melbourne, Australia

ONU, Zoological Museum of Odessa National University, Ukraine

PIN, Paleontological Institute, Moscow, Russia

RBINS, Royal Belgian Institute of Natural Sciences, Brussels, Belgium

SBAER, Inventory of Superintendency of Cultural Heritage of Emilia Romagna Region, Piacenza, Italy

SMNS, Staatliches Museum für Naturkunde, Stuttgart, Germany

SPMI, Mining Institute, St. Petersburg, Russia

TNU, Taurida National University, Simferopol, Ukraine

UCMP, Museum of Paleontology, University of California, Berkeley CA, USA

UL, University of Lisbon, Portugal

USNM, US Museum of Natural History, Smithsonian Institute, Washington DC, USA

ZIRM, Zoological Institute of Republic of Moldova, Chisinău, Moldova

ZMA, Zoological Museum, University of Amsterdam, Netherlands

ZMMU, Zoological Museum of Moscow State University, Russia
