# Supplementary material for: From Problem Taxa to Problem Solver: A New Miocene Family, Tranatocetidae, Brings Perspective on Baleen Whale Evolution
Source: PLoS One. 2015 Sep 2;10(9):e0135500. doi: 10.1371/journal.pone.0135500 (PMC4558012; doi:10.1371/journal.pone.0135500)
Supplement: S2 Appendix — (DOC) [file pone.0135500.s002.doc]

**S2 Appendix List of the specimens used in the analyses**

*Georgiacetus vogtlensis*: GSM 350 (Hulbert et al., 1998)

*Dorudon atrox*: Uhen, 2004

*Eomysticetus whitmorei*: ChM PV4253 (Geisler and Sanders, 2002)

*Aglaocetus patulus*: USNM 23690

*Archaebalaenoptera castriarquati*: SBAER 240536 (Bisconti, 2007)

“*Aulocetus*” *latus*: UL 2

*Balaena mysticetus*: CU CN 1x, 38

*Balaenella brachyrhynus*: NMB 42001 (Bisconti, 2005)

*Balaenoptera acutorostrata*: CU CN 2x, 14x, 247, 1096, 1274, 1275; ZMMU 171919

*Balaenoptera musculus*: CU CN 19x, 38, 29

*Brandtocetus chongulek*: TNU Skull A, TNU Skull 2; TNU Skull 4; ?*Brandtocetus chongulek* TNU X1

*Caperea marginata*: ONU n/n; NMV C28531; Fordyce and Marx, 2013

*Cephalotropis* spp.: *Cephalotropis coronatus*, 9352; *Cephalotropis nectus*, UL 3.

“*Cetotherium*” *megalophysum*: USNM 10593; El Adli et al., 2014

*Cetotherium rathkii*: PIN 1840/1

*Cetotherium riabinini*: NMNH-P 667/1

“*Cetotherium*” *vandelli*: UL 1

*Diorocetus hiatus*: USNM 16783, 23494

*Eschrichtius robustus*: ZMMU 171918, MVZ 125560, MVZ 129689, MVZ 133059

*Eschrichtioides gastaldii*: MGPT PU 13802 (Bisconti, 2008)

*Eubalaena glacialis*: CN 1x, 2x, 4x, 1596

*Eucetotherium helmersenii*: SPMI GG 5,6/114

*Herentalia nigra*: ZMA 5069 (Bisconti, 2014)

*Herpetocetus bramblei*: UCMP 219111

*Herpetocetus transatlanticus*: USNM 182962

*Herpetocetus morrowi*: UCMP 124950; El Adli et al., 2014

“*Idiocetus*” *longifrons*: RBINS CtM 719 / Reg. 769, RBINS CtM 718 / Reg. 1520

*Isanacetus laticephalus*: MFM 28501, 18004 (Kimura and Ozawa, 2002)

*Joumocetus shimizui*: GMNH-PV-2401 (photos kindly provided by Felix Marx)

*Kurdalagonus mchedlidzei*: NMRA 10476

*Mesocetus longirostris*: RBINS CtM 33 / Reg. 401, RBINS CtM 30 / Reg. 400

*Metopocetus durinasus*: USNM 8518

*Miocaperea pulchra*: SMNS 46978 (Bisconti, 2012)

*Mixocetus elysius*: LACM 882

*Morenocetus parvus*: MLP 5-11

*Nannocetus eremus*: UCMP 26502

*Otradnocetus virodovi*: NMG CO 1–90

*Parietobalaena campiniana*: RBINS CtM 399 / Reg. 4018

*Parietobalaena palmeri*: USNM 10668, 16119

?*Parietobalaena securis*: CAS 4579, UCMP 81670

*Pelocetus calvertensis*: USNM 11976

*Peripolocetus vexifiller*: CAS 4370, UCMP 86276

*Pinocetus polonicus*: MZ VIII/Vm 750 (photos kindly provided by Felix Marx)

*Piscobalaena nana*: SMNK-PAL 4050, MNHN SAS 892, 1617, 1618 (Bouetel and de Muizon, 2006)

*Plesiobalaenoptera quarantellii*: MPST 240505

“*Plesiocetopsis hupschii*”: RBINS 664 / Reg. 1240

*Thinocetus arthritis*: USNM 23794

*Titanocetus sammarinensis*: UGGC 9071–9073

*Tranatocetus argillarius*: MGUH VP 2319, MGUH VP 2320

*Uranocetus gramensis*: MSM P813

“*Cetotherium mayeri*”: SPMI GG 44-80/114; CMT 12559/1

Cetotheriidae gen. sp.: ZIRM V28/1
