# Supplementary material for: From Problem Taxa to Problem Solver: A New Miocene Family, Tranatocetidae, Brings Perspective on Baleen Whale Evolution
Source: PLoS One. 2015 Sep 2;10(9):e0135500. doi: 10.1371/journal.pone.0135500 (PMC4558012; doi:10.1371/journal.pone.0135500)
Supplement: S3 Appendix — (DOC) [file pone.0135500.s003.doc]

**S3 Appendix Characters used in phylogenetic analysis**

**Skull**

1. Teeth in adult individuals: present (0); absent (1).
2. Palatal grooves for nutritional flow to baleens: absent (0); present (1).
3. Rostrum, premaxillary-maxillary suture: fused dorsally (0); unfused (1).
4. Rostrum, dorsoventrally arched: not arched (0); moderately arched (1); strongly arched (2).
5. Rostrum, lateral border of maxilla: (0) concave; (1) straight; (2) continuously convex; (3) divided into a short posterior part subparallel to the sagittal plane and a longer straight anterior part converging on the tip of the rostrum.
6. Premaxilla, portion anterior to nasal opening in dorsal view: narrows or remains the same width anteriorly (0); widens at anterior end (1).
7. Premaxilla, ascending (posterior) process: no external contact with frontal (0); premaxilla approximates or contacts frontal.
8. Premaxilla, position of posterior margin of ascending process: anterior to supraorbital process of frontal (0); in line with supraorbital process of the frontal (1); posterior to supraorbital process of frontal (2).
9. Nasal, position of anterior margin: well anterior to antorbital notch (0); approximately in line with antorbital notch (1); well posterior to antorbital notch (2).
10. Premaxillae, dorsal exposure of posterior margin: exposed dorsally forming a transverse line with the posterior ends of nasals and maxillae (0); constricted by ascending processes of maxillae (1); dorsally overridden by ascending processes of maxillae (2).
11. Nasal, position of posterior margin: anterior to supraorbital process of the frontal (0); in transverse line with anterior half of supraorbital process (1); in line with posterior half of supraorbital process or with postorbital process of frontal (2); posterior to postorbital process (3).
12. Nasal, shape: rectangular or trapezoid, longer than wider (0); wedge-shaped, triangular or rhomboid (1).
13. Maxilla, ascending process: absent (0); short, triangular or squared off (1); long, pointed or rounded; (2); long, squared off (3).
14. Maxilla, antorbital process: absent (0); present, constitutes up to half the posterior maxillary width (1); present, constitutes more than half posterior maxillary width (2).
15. Maxilla, posteriormost edge of the posteromedial corner or ascending process: (0) slightly anterior to or in line with anterior half of supraorbital process of frontal; (1) in line with posterior half of supraorbital process or with postorbital process of frontal; (2) posterior to postorbital process; (3) well anterior to anterior edge of orbit.
16. Maxilla/frontal suture: maxilla overrides anteromedial corner of supraorbital process of frontal (0); maxilla almost completely overlays frontal: the frontal is exposed on the vertex as a short area (1); maxilla completely overrides frontal and contacts parietal or supraoccipital (2).
17. Frontal, direction of the anterior border of the supraorbital process of the frontal: laterally (0); anterolaterally (1); posterolaterally (2).
18. Frontal, shape of the supraorbital process: about as wide (lateromedially) as long (anteroposteriorly) (0); about twice as wide as long (1); noticeably more than twice as wide as long (2).
19. Frontal, shape of supraorbital process in dorsal view: medial portion as long (anteroposteriorly) as lateral (0); medial portion distinctly shorter than lateral, supraorbital process has a triangular outline (1); medial portion longer than lateral (2).
20. Frontal, posterior border of supraorbital process: concave (0); straight (1).
21. Frontal, supraorbital processes in anterior or posterior view; horizontal (0); gradually slope lateroventrally away from vertex of skull (1); abruptly depressed at base to a level noticeably below that of dorsal surface of interorbital region (2)
22. Intertemporal constriction: longer than wide (0); wider than long (1).
23. Temporal fossa: longer than wide (0); wider than long (1).
24. Parietal/interparietal, exposure on cranial vertex: clearly exposed on the vertex (0); exposed on the vertex as a short area or closely approximating the vertex (1); excluded from the vertex (2).
25. Sagittal crest: present (0); absent (1).
26. Parietal-frontal suture, anterior wing: anterior wing absent (0); anterior wing present (1).
27. Squamosal, transverse width of exposed portion of squamosal lateral to exoccipital: (0) intermediate width, width between 10 and 34% the distance between sagittal plane and lateral edge of exoccipital; (1) wide, width between 35 and 49% of that distance; (2) very wide, width 50% or more of that distance; (3) narrow, exposed portion of squamosal <10% of that distance
28. Squamosal cleft: absent (0); present (1).
29. Squamosal, zygomatic process: long and slender (0); long and high (1); short, triangular in ventral view (2); not protruding anteriorly (3).
30. Squamosal prominence: forms a dorsal projection on the crest delimiting the lateral or posterolateral edge of the squamosal fossa (0); absent (1).
31. Squamosal, postglenoid process: as wide, as the base of the zygomatic process (0); narrower than the base of the zygomatic process (1)
32. Squamosal, posterior meatal crest forms a lateral projection on the posterolateral side of the postglenoid process: absent (0); present (1).
33. Squamosal, postglenoid process in lateral view: subvertical to anteriorly projecting (0); vertical (1); subvertical to posteriorly projecting (2).
34. Squamosal, postglenoid process in posterior view: ventrally oriented (0); ventrolaterally oriented (1); ventromedially oriented (2).
35. Squamosal, postglenoid process in ventral view: roughly transverse to the sagittal axis of the skull (0); twisted clockwise on the left side and anticlockwise on the right side (1).
36. Squamosal, orientation of the glenoid fossa: directed anteriorly (0); directed ventrally (1); directed anteromedially (2).
37. The anterior extension of the supraoccipital: posterior to temporal fossa (0); in line with temporal fossa (1); in line with supraorbital process of frontal (2); anterior to supraorbital process (3).
38. Occipital shield, shape of anterior margin: rounded or blunt (0); triangular, sharp (1).
39. Occipital shield, shape of lateral margin of nuchal crest in dorsal aspect: convex (0); straight (1); sigmoidal, anteriorly concave (2).
40. Occipital shield, external occipital crest: present (0), absent (1) (Fordyce and Marx, 2013).
41. Occipital shield, bent dorsoventrally: no (0), yes (1)
42. Exoccipital, paroccipital process, in dorsal or ventral view: well posterior to postglenoid process (0), approximately parallel with postglenoid process, medially to it (1); well anterior to postglenoid process (2)
43. Exoccipital, paroccipital process, in dorsal or ventral view: well anterior to occipital condyles (0); approximately parallel with occipital condyles (1); well posterior to occipital condyles (2)
44. Basioccipital crest: narrow transversely (0); massive, square, or triangular (1); massive, longer than wide (2); wing-like (3).
45. Palatines, anteriormost margin: exposed at midline (0); exposed laterally (not at midline) (1).
46. Palatines, posterior extension: extended to internal nares (0); extended to slightly underlap the pterygoids (1); long underlap of pterygoids nearly reaching the posterior border of the inferior lamina of the pterygoid fossa (2).
47. Vomer, forms a prominent ventral keel on the palate: no (0); yes (1).
48. Pterygoid, extensive exposure in ventral aspect: yes (0); no (1).
49. Auditory meatus sulcus: broad and short (0); long and narrow (1).

**Periotic**

1. Lateral projection of the anterior process of the periotic body: absent or small (0); large (1); hypertrophied (2).
2. Pars cochlearis bulges ventral to fenestra rotunda: no (0); yes (1).
3. Posterior cochlear crest: absent or small (0); present and extending directly posteriorly (1); large and posteroventrally extending (2).
4. Pars cochlearis medially compressed and receding dorsomedially, in posterior view: no (0); yes (1).
5. Dorsal surface of pars cochlearis: flat (0); sharp edges surround the dorsal foramina (1); concave and dorsomedially facing (2); like state 2, but foramina extending in siphon (3).
6. Posterior process(es) of the tympanoperiotic: short, less than the length of the periotic body (0); approximately as long as the periotic body or longer (1); short and thick, with a flat or convex lateral surface (2); hypertrophied (3).
7. Shape and size of the distal surface of the posterior process of the tympanoperiotic exposed at the posterolateral wall of the skull: lateral exposure absent (0); small (height < 25% of height of the paroccipital process), irregular (1), large (height > 50% of height of the paroccipital process), quadrangular (2), large, triangular (3), large, oval or round (4), medium-sized, rhomboid or pentagonal, anteriorly directed (5).
8. The neck of the posterior process of the periotic: transversely constricted (0); transversely and dorsoventrally constricted (1); absent (2).
9. Periotic, groove for tensor tympani muscle: present and well defined (0); absent or poorly developed (1).
10. Periotic*,* suprameatal fossa: present (0); absent (1).
11. Periotic*,* arrangement of endolymphatic and perilymphatic foramina: separate, with thin bony septum that is anteroposteriorly oriented (0); en echelon, divided by a thin bony septum that is dorsoventrally oriented (1).
12. Shape of the pars cochlearis: the width (anteroposterior) is approximately twice the height (dorsoventrally) (0); the width is approximately the same as the height (1); the height is twice the width or more (2).
13. The facial nerve canal opening (VII), the internal auditory meatus and the perilymphatic foramen dorsally on the pars cochlearis are aligned: no (0); yes (1).
14. Shape and size of the endolymphatic foramen: small (0); approximately the size of the internal auditory meatus, slit-shaped (1).
15. The mallear fossa groove: not distinct (0); distinct, long, and anteriorly directed (1); distinct and posteriorly directed (2).
16. Mallear fossa: shallow and/or broad (0); deep and narrow (1).

**Tympanic bulla**

1. Bulla bilobed posteriorly: yes (0); no (1).
2. Shape of the anterior end of bulla in medial view: narrowed (0); oval (1); squared (2).
3. Shape of the bulla in ventral view: ovoid or rectangular, the maximum width in the medial portion (0); rhomboid or trapezoid, the maximum width in the anterior portion (1); posterolateral end of bulla inflated, the maximum width in the posterior portion (2); anterolateral end of bulla inflated, the maximum width in anterior or medial portion (3).
4. Bulla in medial view: short, width is > 40% of the length (0); long (1).
5. Deep lateral furrow: no (0); yes (1).
6. The sigmoid process of the bulla is long and the area with and around the process is protruding: no (0); yes (1).
7. Tympanic bulla, anterolateral ridge or shelf: absent (0); present (1).
8. Tympanic bulla, conical process height in proportion to length narrow or absent (0); wide, width greater than 40% of length (1).
9. Tympanic bulla, dorsal involucral surface in medial view: relatively straight or slightly curved (0); markedly sinuous and strongly concave (1).
10. Tympanic bulla, squared posterior portion of medial margin of main ridge: absent (0); present (1).
11. Tympanic bulla, length of anterior lobe: long, greater than one-third of total length of bulla (0); short, one-third or less than total length of bulla (1).
12. Tympanic bulla, main ridge and involucral ridge: extend parallel with one another (0); convergent at posterior end (1); convergent at anterior end (2).

**Mandible**

1. Neck in dorsal aspect: straight neck (0); recurved neck (1).
2. Shape of the mandibular condyle: small, triangular (0); broad and high, taking up the entire posterior end of the mandible (1); narrow, articular surface oblique to the longitudinal axis of the ramus, rather than at a straight angle (2); spherical (4).
3. Orientation of articular surface of mandibular condyle: posterior (0); posterodorsal (1); dorsal with the condyle being confluent with a dorsoventrally expanded angular process (2); dorsal with the condyle being larger than and clearly offset from the angular process (3).
4. Mandibular foramen size compared to neck of mandible: large, maximum foramen height >50% maximum height of neck (0); small, maximum foramen height <50% maximum height of neck (1).
5. Relative position of anterior border of mandibular foramen: anterior to coronoid process (0); In line with the coronoid process (1); posterior to coronoid process (2).
6. Subcondyle furrow: absent (0); present and open medially (1); present and open dorsally (2).
7. Shape of the coronoid process: triangular, long and high (0); triangular, long and low (1); very low (crest-like) or absent (2); double, high or very low (3).
8. Position of coronoid process: located relatively close to mandibular condyle (0); located relatively far anterior to mandibular condyle (1).
9. Position of coronoid process, posterior margin of the base: (0) dorsal to the condyle; at the same level or ventral to the condyle (1)
10. Position of angular process: located below the condyle or slightly anterior (0); projects posteriorly to a level posterior to the condyle (1).
11. Postcoronoid elevation: absent (0); present (1).

**Postcranial elements**

1. Cervicals 3–6, ends of upper and lower transverse processes: fused, the transverse foramen is formed (0); unfused (1).
2. Number of thoracic vertebrae: 14 or less (0), >14 (1).
3. Number of lumbar vertebrae: < 9 (0), 9-14 (1), >14 (2).
4. Sternum, xiphioid process: present (0); absent (1).
5. Acromion and coracoid processes of scapula: both present (0); coracoid process absent (1); acromion or both processes absent (2).
6. Scapula proportions: anteroposterior (proximodistal) diameter is as long as the transverse diameter or longer (0); anteroposterior diameter is twice shorter as the transverse diameter or longer (1); anteroposterior diameter is shorter than the half of the transverse diameter (2).
7. Scapula, glenoid fossa: in the middle between dorsal and ventral margins (0); distinctly closer to the dorsal margin (1).
8. Humerus: longer than radius and ulna (0); same length (1); shorter than radius and ulna (2).
9. Deltoid crest of humerus: present as a distinct crest (0); absent or reduced to a variably developed rugosity (1).
10. Humeral head: small, its proximodistal projection is one-third of the humerus length or shorter (0); large, at least 40% of the humerus length (1)
11. Orientation of humeral head in lateral view: posterodorsal; the head is hemispherical in lateral view (0); dorsal (1); posterolateral or dorsolateral; the head is spherical in lateral view (2)
12. Distal portion of humerus in lateral view: distal epiphysis narrower than shaft (0); distal epiphysis flared compared to shaft (1).
13. Olecranon process: present as a distinct process (0); absent (1).
14. Manus: 5 digits (0); 4 digits (1).

Additional references

Bisconti M. 2005 Skull morphology and phylogenetic relationships of a new diminutive balaenid from the lower Pliocene of Belgium. *Palaeontology* **48,** 793-816.

Bisconti M. 2007 A new basal balaenopterid whale from the Pliocene of northern Italy. *Palaeontology* **50,**1103-1122.

Bisconti M. 2008 Morphology and phylogenetic relationships of a new eschrichtiid genus (Cetacea: Mysticeti) from the early Pliocene of northern Italy. *Zool. J. Linnean Soc.***153**, 161-186.

Bisconti M. 2012. Comparative osteology and phylogenetic relationships of Miocaperea pulchra, the first fossil pygmy right whale genus and species (Cetacea, Mysticeti, Neobalaenidae). *Zool. J. Linnean Soc.***166**, 876-911.

Hulbert RC, Petkewich RM, Bishop GA, Bukry D, Aleshire DP. 1998 A new middle Eocene protocetid whale (Mammalia: Cetacea: Archaeoceti) and associated biota from Georgia. *J. Paleontol.* **72,** 907-927.

Kimura T, Ozawa T. 2002 A new cetothere (Cetacea: Mysticeti) from the early Miocene of Japan. *J. Vertebr. Paleontol.* **22,** 684-702.

Uhen MD. 2004 Form, Function, and Anatomy of Dorudon atrox (Mammalia, Cetacea): An Archaeocete from the Middle to Late Eocene of Egypt. *Univ. Michigan Papers Paleontol.* **34**, 1-222.
