# Supplementary material for: From Problem Taxa to Problem Solver: A New Miocene Family, Tranatocetidae, Brings Perspective on Baleen Whale Evolution
Source: PLoS One. 2015 Sep 2;10(9):e0135500. doi: 10.1371/journal.pone.0135500 (PMC4558012; doi:10.1371/journal.pone.0135500)
Supplement: S1 File — (PDF) [file pone.0135500.s004.pdf]

Gmail by Google

Pavel Gol'din <pavelgoldin412@gmail.com>

publication of your photos

Felix G. Marx <felix.marx@otago.ac.nz> Wed, Jun 3, 2015 at 7:46 AM
To: Pavel Gol'din <pavelgoldin412@gmail.com>

Dear Pavel,

no problem - I hereby give you permission to publish my photos showing Piscobalaena (vertex) and Caperea (bulla) under a CC BY licence for your upcoming article. Please make sure the repositories and specimen numbers are properly cited.

Great to hear the article is moving forward. When do you expect it to be published?

[redacted]

All the best,

Felix

Felix G. Marx PhD
JSPS Postdoctoral Research Fellow
Division of Biotic Evolution/ Department of Geology and Palaeontology
National Museum of Nature and Science
4-1-1 Amakubo, Tsukuba, Ibaraki 305-0005, Japan; tel. +81 (0)29-853-8263

From: Pavel Gol'din [pavelgoldin412@gmail.com]
Sent: 03 June 2015 09:36
To: Felix G. Marx; Felix G Marx
Subject: publication of your photos

[Quoted text hidden]
